# Fatal Lead Toxicity From Ayurvedic Supplements in a Patient With Parkinson’s Disease

**DOI:** 10.7759/cureus.90515

**Published:** 2025-08-19

**Authors:** Agnieszka Gryguc-Saxanoff

**Affiliations:** 1 Hospital Medicine, Ronald Reagan University of California Los Angeles Medical Center, Los Angeles, USA

**Keywords:** altered mental status evaluation, ayurvedic medicine, chronic lead poisoning, elderly population, parkinson's disease, rare cause of altered mental status

## Abstract

Lead poisoning is rare in adults, particularly when the source of exposure is not immediately apparent. This case describes a 76-year-old woman with Parkinson’s disease who presented with altered mental status after taking Ayurvedic supplements for several weeks. Clinical evaluation revealed a blood lead level greater than 90 µg/dL (measured after her supplement use was identified) and abdominal imaging that demonstrated radiopaque material throughout the colon, findings consistent with lead ingestion. Although chelation therapy was initiated promptly, her condition worsened due to progressive neurologic decline and the development of secondary infections. After discussions with her family, the focus of care was transitioned to comfort measures. This case highlights the importance of obtaining a thorough medication history, including over-the-counter and alternative therapies, both on admission and throughout hospitalization. It also underscores the serious risks associated with heavy metal exposure, particularly in patients with preexisting neurologic disorders, and the need for early, guideline-based management.

## Introduction

Lead exposure in adults has declined markedly due to regulatory reforms; however, traditional and alternative medicines remain underrecognized sources of heavy metal toxicity. Ayurvedic medicine, an ethnomedicine originating in India, sometimes incorporates mineral and metal preparations such as *Naga bhasma *(a processed lead compound), *Parada *(elemental mercury), and *Manahshila *(arsenic sulfide), either intentionally for perceived therapeutic benefit or inadvertently due to contamination during manufacturing [1-3]. Analyses have found detectable levels of heavy metals in up to 25% of Ayurvedic supplements sold online [2,3].

Lead poisoning in adults can present with a broad spectrum of clinical features depending on the level and duration of exposure. At blood lead levels above 40 µg/dL, symptoms may include fatigue, irritability, and mild cognitive changes. Levels exceeding 70-80 µg/dL are typically associated with severe abdominal pain (“lead colic”), anemia, and encephalopathy, while levels over 100 µg/dL can cause seizures, coma, and death [4]. Chronic low-level exposure may result in subtle cognitive decline, anemia, and neuropathy, whereas acute high-level exposure often produces rapid, severe manifestations.

Patients with chronic neurologic illnesses, such as Parkinson’s disease, may be particularly vulnerable to the neurotoxic effects of lead. Lead has been linked to oxidative stress, mitochondrial dysfunction, and alpha-synuclein aggregation, mechanisms implicated in the pathogenesis of Parkinson’s disease [5-8]. Public health investigations have also documented clusters of lead poisoning in the United States linked to Ayurvedic medications [6].

This clinical scenario illustrates the diagnostic challenges of lead toxicity, the correlation between symptom severity and blood lead concentration, and the importance of culturally sensitive history-taking and early toxicology evaluation.

## Case presentation

A 76-year-old woman with a six-year history of Parkinson’s disease was brought to the emergency department with acute confusion, preceded by several weeks of worsening lethargy, poor oral intake, and vague abdominal discomfort. At baseline, she had been alert, oriented, and largely independent in activities of daily living (ADLs), ambulating without supportive devices.

On arrival, her vital signs were stable. Physical examination revealed bradykinesia, rigidity, and altered mentation, with a Glasgow Coma Scale (GCS) score of 11. No focal neurologic deficits were observed. Initial laboratory studies showed mild hyponatremia (serum sodium: 129 mmol/L) and normocytic anemia (hemoglobin: 10.2 g/dL), with an otherwise unremarkable metabolic panel. A urine culture identified *Escherichia coli*, for which antibiotic therapy was initiated. Hyponatremia was attributed to poor oral intake and was corrected with supportive measures.

**Figure 1:**
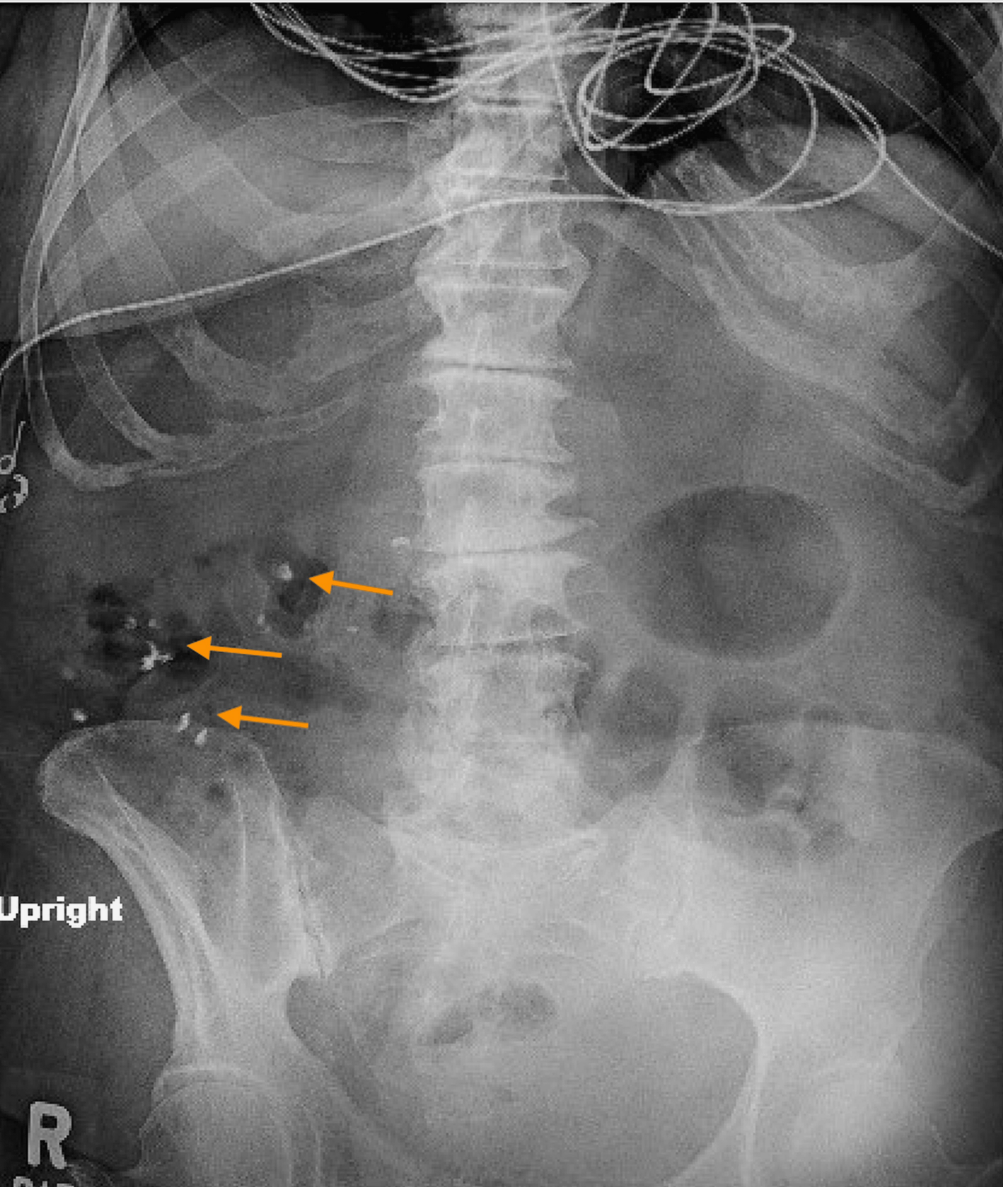
Portable kidney, ureter, and bladder X-ray showing punctate radiopaque material throughout the colon (arrows)

Despite resolution of infection and normalization of electrolytes, her mental status remained impaired. A detailed interview with her family revealed that ten weeks before admission, she had begun taking multiple Ayurvedic supplements for “general wellness” (the exact formulations were unknown), prompting heavy metal testing. A venous blood lead level was greater than 90 µg/dL, a critical range associated with high risk of encephalopathy, anemia, and abdominal pain (Table 1) [4]. An abdominal radiograph (kidney, ureter, and bladder) revealed punctate radiopaque material in the right lower quadrant, consistent with retained lead-containing particles (Figure 1).

**Table 1:** Laboratory findings and reference ranges

Laboratory test	Patient result	Reference range	Interpretation
Blood lead level	>90 µg/dL	<5 µg/dL	Severely elevated
Hemoglobin	10.2 g/dL	12.0-16.0 g/dL (female)	Mild anemia
Sodium	129 mmol/L	135-145 mmol/L	Mild hyponatremia
Metabolic panel	Unremarkable	Not applicable	Within normal limits

Her constipation and anemia were considered likely secondary to lead exposure, and iron studies were recommended, as iron deficiency can increase gastrointestinal lead absorption [4]. A head CT scan showed no acute abnormalities, further supporting the suspicion that her presentation was primarily due to lead toxicity. She was transferred from the medical floor to the intensive care unit and started on guideline-recommended chelation therapy with intramuscular dimercaprol combined with oral calcium disodium ethylenediaminetetraacetic acid [4]. This regimen is indicated in cases of severe toxicity or encephalopathy to rapidly reduce circulating lead levels.

Despite modest biochemical improvement, her neurologic function deteriorated to a GCS of 3, and she developed aspiration pneumonia and a catheter-associated urinary tract infection, both of which progressed to sepsis. After a palliative care consultation and discussion with her family, she was transitioned to comfort care and died shortly thereafter.

The unifying diagnosis was severe lead encephalopathy secondary to Ayurvedic supplement ingestion, reached after other potential causes of encephalopathy, including metabolic disturbances, structural brain lesions, and infection, were either excluded through diagnostic testing or addressed without improvement in her neurologic status. The persistently elevated blood lead level, imaging evidence of retained lead-containing material, and symptom onset shortly after beginning the supplements all supported this diagnosis.

## Discussion

Although uncommon, adult lead toxicity remains a significant concern in the era of global supplement access. Ayurvedic medicines have repeatedly been linked to heavy metal contamination [1-3]. Their marketing as “natural” or “immune-boosting” may promote use and foster a false sense of safety. Many patients, particularly older adults or those from cultural backgrounds where such products are commonly used, may not disclose supplement use unless specifically asked. Table 2 lists examples of Ayurvedic formulations that have been found to contain heavy metals, either intentionally or through contamination [2,3].

**Table 2:** Examples of Ayurvedic remedies with documented heavy metal content

Product name	Documented metals	Country of origin	Purpose of use	Cited sources
Mahayograj Guggulu	Lead, mercury	India	Arthritis, joint pain	[2,6]
Navratna Rasa	Mercury, arsenic	India	General vitality, immunity	[2,6]
Trivanga Bhasma	Lead, tin, zinc	India	Urogenital disorders	[2,6]
Rasa Parpati	Mercury, sulfur	India	Gastrointestinal complaints	[2,6]

In this case, targeted questioning revealed the exposure and facilitated timely testing for heavy metals. Lead toxicity in adults presents with variable manifestations, with severity depending on both dose and duration of exposure. Common features include anemia, abdominal pain, constipation, and neuropsychiatric changes [4]. These symptoms are nonspecific and can mimic the progression of Parkinson’s disease [5]. Acute, high-level exposure often causes rapid symptom onset, whereas chronic low-level exposure allows gradual accumulation in bone and soft tissues, leading to more insidious neurologic and hematologic effects [4]. Lead is stored primarily in bone (90-95% of total body burden) and may be mobilized during periods of increased bone turnover, further elevating blood levels [4]. Guideline-based management in adults includes removal of exposure, administration of oral succimer, and chelation therapy (Table 3) [4].

**Table 3:** Medical management of adult lead exposure EDTA, ethylenediaminetetraacetic acid

Blood lead level	Clinical scenario	Recommended management
<45 µg/dL	Asymptomatic	Remove exposure; no chelation
45-69 µg/dL	Symptomatic	Consider oral succimer (dimercaptosuccinic acid)
≥70 µg/dL or any severe symptoms	Severe toxicity	Hospitalize; initiate parenteral chelation with calcium disodium EDTA ± dimercaprol
Any level with encephalopathy	Lead-induced encephalopathy	Dimercaprol plus calcium disodium EDTA

In this case, the combination of altered mental status, anemia, and abdominal radiopaque material was consistent with severe lead colic and encephalopathy. The patient’s blood lead level (>90 µg/dL) exceeded the threshold for high-risk neurotoxicity, warranting immediate combination chelation therapy. Unfortunately, once severe encephalopathy develops, chelation can lower circulating lead levels but cannot reverse established neuronal injury. From a public health standpoint, the absence of pre-market safety evaluation for most imported supplements in the United States [2,3] allows contaminated products to remain widely available. Clinicians should routinely inquire about herbal, imported, and over-the-counter supplements, particularly in patients with unexplained neurologic, gastrointestinal, or hematologic abnormalities.

## Conclusions

Lead toxicity from alternative medicines remains a preventable but underrecognized cause of severe morbidity and mortality. Although regulatory measures have reduced industrial and environmental lead exposure, traditional and imported remedies continue to pose a significant risk. Clinicians should remain aware of symptom-level correlations, current treatment thresholds, and the importance of obtaining a comprehensive medication history, including culturally specific and imported remedies. Early recognition and intervention are critical to preventing irreversible harm, especially in patients with preexisting neurologic disease.
